# Correlation of the combined symptom and medication score with quality of life, symptom severity and symptom control in allergic rhinoconjunctivitis

**DOI:** 10.1002/clt2.12191

**Published:** 2022-10-05

**Authors:** Binoy Palathumpattu, Ursula Pieper‐Fürst, Cengizhan Acikel, Hacer Sahin, Silke Allekotte, Jaswinder Singh, Mark Hess, Angelika Sager, Thomas Müller, Ralph Mösges

**Affiliations:** ^1^ Institute of Medical Statistics and Computational Biology Faculty of Medicine University of Cologne Cologne Germany; ^2^ ClinCompetence Cologne GmbH Cologne Germany; ^3^ LETI Pharma GmbH Ismaning Germany

**Keywords:** allergen‐specific immunotherapy, allergic rhinoconjunctivitis, clinical endpoint, combined symptom and medication score, correlation

## Abstract

**Background:**

The European Academy of Allergy and Clinical Immunology recommended the Combined Symptom and Medication Score (CSMS) as primary endpoint in clinical trials on allergen‐specific immunotherapy (AIT) in allergic rhinoconjunctivitis. Here, the correlation between the CSMS and the validated standardised Rhinoconjunctivitis Quality of Life Questionnaire (RQLQ(S)), Rhinitis Control Assessment Test (RCAT) and Visual Analogue Scale (VAS) was analysed.

**Methods:**

Two prospective, multicentre, non‐interventional studies on tree pollen, grass pollen and house dust mite allergic patients were performed. The first study comprised 167 patients receiving AIT (AIT population), and the second included 56 patients treated with symptomatic medication only (control population). For up to two seasons (pollen)/exposure periods (house dust mites), participants documented their symptoms and medication intake in a CSMS diary, including VAS. In addition, the standardised RQLQ(S) and the RCAT were completed during study visits.

**Results:**

Comparison between CSMS and RQLQ(S) revealed a positive correlation in the AIT population (*r* = 0.426) and in the control population (*r* = 0.569). For CSMS and RCAT, a negative correlation with *r* = −0.409 (AIT) and *r* = −0.547 (control) was shown. Positive correlation between CSMS and VAS was also demonstrated with *r* = 0.585 (AIT) and *r* = 0.563 (control).

**Conclusion:**

These results support the assumption that the CSMS correlates with quality of life, symptom severity and symptom control on the one hand, while the moderate strength of correlations on the other hand mirrors distinctions of the CSMS compared to the assessments used here.

## 1 INTRODUCTION

Allergic rhinoconjunctivitis (ARC) is a predominantly eosinophil‐mediated inflammation of the nasal mucosa and conjunctiva.^1^ It is one of the most common allergic disorders, with varying estimates of prevalence and manifestations across different parts of the world.^1^, ^2^, ^3^, ^4^, ^5^ The burden of ARC can affect patients' social life and educational or job performance, and is associated with high economic costs.^6^, ^7^, ^8^, ^9^, ^10^ Besides allergen avoidance, there are two principal pharmaceutical approaches to treat ARC. First, in conventional pharmacotherapy, antihistamines, antileukotrienes, corticosteroids or monoclonal antibodies are used to reduce or control the symptoms.^11^ Secondly, allergen‐specific immunotherapy (AIT) is the only disease modifying causal therapy being available aiming to induce long‐term tolerance to allergens.^11^, ^12^, ^13^ In AIT, allergens are presented to the immune system, either by subcutaneous injection (subcutaneous immunotherapy ‐ SCIT) or sublingually (sublingual immunotherapy ‐ SLIT).^12^ Randomised clinical trials assessing AIT in ARC show clinical and methodological heterogeneity on the defined endpoints, making comparisons difficult and to date commonly accepted standards have not been established.^13^, ^14^, ^15^ Widely used instruments which have already been proven to reflect the extent of ARC associated impact on the quality of life, symptom control and impairment by symptoms, respectively, include the Rhinoconjunctivitis Quality of Life Questionnaire (RQLQ),^16^, ^17^ the Rhinitis Control Assessment Test (RCAT)^18^, ^19^, ^20^ and Visual Analogue Scales (VAS).^21^, ^22^, ^23^, ^24^ To standardise the primary outcome in clinical trials for proof of efficacy in AIT and in line with the European Medicines Agency guideline in the development of products for specific immunotherapy, the European Academy of Allergy and Clinical Immunology (EAACI) recommended the Combined Symptom and Medication Score (CSMS).^13^, ^25^ The CSMS reflects the sum of daily Symptom Score (dSS) and daily Medication Score (dMS) comprising the rating of 6 symptoms and use of symptomatic medication.^13^ In the present study, we performed a comprehensive correlation analysis on CSMS outcomes versus the standardised RQLQ, the RCAT and VAS in order to contribute to the discussions on its applicability as primary endpoint for clinical studies on AIT.

## 2 METHODS

### 2.1 Study design

We performed two prospective multicentre non‐interventional studies based on a common master protocol. The first was an observational study in accordance with Section 4 subsection 23 sentence three of the German Medicinal Product Act,^26^ comprising patients (“AIT population”) treated with pre‐seasonal or perennial subcutaneous AIT with depigmented allergoids.^27^ The second was a prospective data acquisition with patients (“control population”) taking symptomatic medication only. In up to two observation periods (September 2018–July 2019 and September 2019–July 2020) the participants were requested to complete a CSMS diary, including VAS demonstrating the overall impairment due to ARC symptoms, for at least 30 days during the season (pollen) or exposure period (house dust mites, especially from September to December). In addition, the standardised RQLQ (RQLQ(S); at visits 1–3 and 4–6, if applicable) and the RCAT (at visit 2 and 5, if applicable) were filled in at the study centres. The timing of the visits in relation to the observation period is given in Table 1.

**TABLE 1:** Overview of visits and assessments

Observation period	Sep 2018–Jul 2019 (PS 2019, EP 2018)			Sep 2019–Jul 2020 (PS 2020, EP 2019)		
House dust mites exposure	Sep–Dec^c^			Sep–Dec^c^		
Tree pollen season	Mar–May			Mar–May		
Grass pollen season	May–Jul			May–Jul		
Visits	V1	V2	V3	V4^a^	V5^a^	V6^a^
				V1^b^	V2^b^	V3^b^
	Prior to/start of PS/EP	Peak of PS/EP	End of/after PS/EP	Prior to/start of PS/EP	Peak of PS/EP	End of/after PS/EP
Start of CSMS diary, including VAS	×			×		
End of CSMS diary, including VAS			×			×
RQLQ(S)	×	×	×	×	×	×
RCAT		×			×	

Abbreviations: CSMS, Combined Symptom and Medication Score; EP, Exposure period (house dust mites); PS, Pollen season; RCAT, Rhinitis Control Assessment Test; RQLQ(S), Standardised Rhinoconjunctivitis Quality of Life Questionnaire; VAS, Visual Analogue Scales.

^a^ AIT population patients with two assigned observations.

^b^ AIT population patients with single observation period and control population patients.

^c^ Designated observation period.

### 2.2 Ethic compliance

The studies complied with the Declaration of Helsinki and the Good Clinical Practice principles of the International Conference on Harmonisation. Ethical approval was given by the Ethics Committee of the Medical Faculty of the University of Cologne (internal references for the AIT study and control study: 18–120 and 19–1347, respectively). According with the German Medicinal Products Act, the responsible authorities were notified about the AIT study prior to the beginning. Data protection was ensured in accordance with applicable German guidelines. Informed consent was obtained from patients. Both studies were registered on the clinicaltrials.gov platform under the following identifiers: NCT03850626 (AIT) and NCT04071249 (control).

During the AIT study, there were no cases of fatality, anaphylaxis or adverse reactions with the need for adrenaline injection. Adverse reactions were mainly local reactions at the injection site. Furthermore, systemic reactions up to grade 1 according to WAO criteria^28^ such as cough, fatigue, headache and dyspnoea occurred. All adverse reactions were fully resolved.

### 2.3 Study populations

Eligible patients were adults and children/adolescents aged ≥12 years, suffering from allergies to house dust mites (HDM), tree pollen (TP) or grass pollen (GP) and consequently from rhinitis, conjunctivitis or rhinoconjunctivitis. Individuals with additional allergic asthma were also eligible. In both studies, treatment had already begun, or the decision for therapy had already been made irrespective of study participation. Randomisation, stratification or matching was not performed. The application of AIT and/or symptomatic medication strictly followed therapeutic indication and purposes given in the summary of product characteristics. Observed adverse events in patients treated with AIT were reported by the investigators, who were physicians specialised in allergology and experienced in the application of AIT.

Patients in the AIT population enrolled between September 2018 and May 2019 were to be observed during two consecutive pollen seasons (PS 2019 and PS 2020) or two exposure periods (HDM, EP 2018 and EP 2019). Since the intended number of subjects was not reached during the first enrolment phase, the enrolment period was prolonged (August 2019–June 2020), allowing the enrolment of new patients for observation during one single pollen season/exposure period (i.e., PS 2020/EP 2019). In the control population, all patients were enrolled between October 2019 and June 2020 and were assigned for a single observation period (PS 2020/EP 2019).

### 2.4 Assessments

An overview of the observation periods and visits, including the questionnaires used, is shown in Table 1.

The RQLQ(S) refers to the previous 7 days, containing 28 questions, covering 7 domains (activities limitation, sleep problems, non‐hay fever symptoms, practical problems, nasal symptoms, eye symptoms and emotional function). A Likert scale from 0 to six is given. The mean of all seven domains reflects overall rhinoconjunctivitis‐associated quality of life (QoL), with a possible result between 0 and 6 and lower scores indicating higher QoL.

The RCAT assesses the extent of rhinitis symptom control for the preceding week. Six items are covered: frequency of nasal congestion, sneezing and watery eyes, sleep disruption (caused by allergy symptoms), activity limitation caused by symptoms and self‐rating of rhinitis symptom control. A 5‐point Likert scale is used to rate each item, yielding a score up to 30. A score ≤21 reflects not well controlled symptoms.

VASs were applied to assess overall impairment by ARC symptoms (overall VAS), specific impairment by nasal symptoms (nasal VAS) and specific impairment by conjunctival symptoms (conjunctival VAS). Rating was done by marking in a scale between “not impairing” (=0) and “very much impairing” (=100).

The CSMS reflects both symptoms and intake of rescue medication equally weighted daily. The higher the score, the higher the impact of ARC. Table 2 gives a detailed overview of how the CSMS is calculated.

**TABLE 2:** Calculation of the Combined Symptom and Medication Score (adapted from Pfaar et al.^13^)

a) Symptom score		
0 = no symptoms		
1 = mild symptoms (clearly present, but minimal awareness; easily tolerated)		
2 = moderate symptoms (definite awareness; bothersome, but tolerable)		
3 = severe symptoms (hardly tolerable; interfere with activities of daily living and/or sleeping)		
Nasal symptoms	Nasal pruritus	0–3
	Sneezing	0–3
	Rhinorrhoea	0–3
	Nasal obstruction	0–3
Conjunctival symptoms	Itchy/red eyes	0–3
	Watery eyes	0–3
(Total) daily symptom score (dSS) = up to 18 points divided by 6		0–3
b) Medication score		
No use of rescue medication		0
Oral and/or topical (eyes/nose) nonsedating H1‐antihistamines (H1A)		1
Intranasal corticosteroids (INS) with/without H1A		2
Oral corticosteroids with/without INS, with/without H1A		3
(Total) daily medication score (dMS)		0–3
c) Combined symptom and medication score CSMS = dSS + dMS		0–6

### 2.5 Data acquisition

The CSMS diary was used for documentation of rhinoconjunctivitis symptoms, symptomatic medication, and impairment by symptoms. It was kept electronically, using the application CSMS+ Diary, including VASs, for mobile devices (Android version 1.022, iOS version 1.1, released by AppCologne GmbH, Cologne, Germany). In 11 cases, the diary was kept on printed forms due to technical reasons. RQLQ(S) and RCAT were filled in on printed forms. During the observation periods, diary data were checked for continuity twice a week. Besides, the diary app reminded the patients of doing their entries daily. The questionnaires were checked for completeness and plausibility. By double data entry and reconciliation, data integrity of all paper‐based question forms and questionnaires was ensured. The data from CSMS+ Diary was exported and merged with the data from the paper‐based question forms. All data were analysed using a patient ID number only. These data were further matched with the information on the pollen season (PS) valid for the respective study location. The start and the end of the PS were defined according to Pfaar et al.^29^ PS started at the first of 5 days (out of 7 consecutive days), each with ≥3 (grass) or 10 pollen/m^3^ (birch) daily and accounting for a sum of ≥30 (grass) or 100 pollen/m^3^ (birch). The end was marked by the last day of series of 5 days, meeting these criteria. Each study site was assigned to a pollen region as defined by Germany's National Meteorological Service (DWD).^30^ Pollen exposure levels associated to the expected daily mean pollen concentration were classified from 0 (no exposure) to 3 (high exposure) as given by the DWD. The exposure period (EP) for house dust mites was defined as the 30 days with worst incidence of symptoms as documented in the patients' diary.

### 2.6 Statistical analysis

For data entry and statistical analysis, the Statistical Package for the Social Sciences Version 25 for Windows (SPSS®, IBM, Armonk, NY, USA) was used. Demographic and baseline characteristics were analysed by descriptive statistics. Continuous variables were described by numbers of valid or missing data, mean and standard deviation. Categorical data were shown as absolute frequency and/or percentage. Pearson coefficient was calculated for the correlation between mean CSMS and RQLQ(S) at V2 (and V5, if applicable), mean CSMS and mean overall VAS, as well as mean CSMS and RCAT at V2 (and V5, if applicable).

## 3 RESULTS

Throughout the entire study duration (September 2018 to August 2020), 223 patients were included in 20 study centres throughout Germany. Of these, 167 belonged to the AIT population and 56 belonged to the control population.

In the AIT population, 31 TP patients, 29 GP patients and 57 HDM patients were assigned for two observation periods. 15 TP patients, 11 GP patients and 24 HDM patients were assigned for a single observation period. 10 patients out of 167 were children/adolescents (1 in the TP, 3 in the GP and 6 in the HDM group). Of 117 patients who were assigned for 2 observations, 91 patients participated in the second observation period and 86 patients (73.5%) completed 6 visits. Regarding the 50 patients assigned for a single observation, 48 completed the requested three visits (96%). In the control population, the majority was included in the GP group. There were 3 adolescents (1 in the GP, two in the HDM group). Out of 56 patients, 54 completed the requested three visits.

### 3.1 Demographic characteristics

Table 3 gives an overview of the demographic characteristics, which were comparable between both populations.

**TABLE 3:** Demographic characteristics

Group		Tree pollen	Grass pollen	House dust mites
Population				
AIT	*N*	46 (27.5%)	40 (24.0%)	81 (48.5%)
	Age (Ø in years) [SD]	43.96 [13.28]	33.73 [11.66]	33.85 [11.85]
	Gender			
	Female	29 (63.0%)	21 (52.5%)	51 (63.0%)
	Male	17 (37.0%)	19 (47.5%)	30 (37.0%)
Control	*N*	8 (14.3%)	33 (58.9%)	15 (26.8%)
	Age (Ø in years) [SD]	38.25 [13.74]	31.61 [13.02]	29.40 [11.67]
	Gender			
	Female	4 (50.0%)	21 (63.6%)	8 (53.3%)
	Male	4 (50.0%)	12 (36.4%)	7 (46.7%)

Abbreviations: Ø, mean; SD, standard deviation.

### 3.2 CSMS data acquisition

In total, 20,300 patient diary entries were collected. Of these, 20,294 (>99.9%) (2018: 1595, 2019: 10,718, 2020: 7981 entries) were suitable for CSMS calculation. For 2020, completeness rate of the patients' diaries was calculated based on a 30‐day pollen period, that is, 30 days in April for tree pollen and 30 days in June for grass pollen, showing data were entered on 81.9% of the days in the tree pollen period and on 83.6% of the days of the grass pollen period.

Mean score values of CSMS/VAS and RQLQ(S)/RCAT are given in Tables 4 and 5, respectively.

**TABLE 4:** Combined symptom and medication score (CSMS) and VAS scores obtained via mobile phone diary application

Pollen season 2019/exposure period 2018		CSMS	VAS overall
TP AIT with 2 observations	*N*	26	26
	Mean [SD]	1.52 [1.00]	31.52 [17.47]
GP AIT with 2 observations	*N*	26	26
	Mean [SD]	1.29 [0.80]	24.90 [20.05]
HDM AIT with 2 observations	*N*	54	54
	Mean [SD]	1.34 [1.00]	36.39 [24.49]

Pollen season 2020/exposure period 2019			
TP AIT with 2 observations	*N*	23	23
	Mean [SD]	1.44 [0.88]	31.42 [20.61]
TP AIT with single observation	*N*	14	14
	Mean [SD]	1.85 [1.09]	38.75 [23.77]
TP Control	*N*	8	8
	Mean [SD]	1.92 [0.94]	31.34 [27.75]
GP AIT with 2 observations	*N*	19	19
	Mean [SD]	1.54 [0,69]	32.07 [20.00]
GP AIT with single observation	*N*	11	11
	Mean [SD]	1.63 [0,80]	35.97 [24.16]
GP Control	*N*	33	33
	Mean [SD]	1.62 [0,88]	32.52 [20.41]
HDM with 2 observations	*N*	46	46
	Mean [SD]	1.11 [0,88]	28.81 [23.06]
HDM AIT with single observation	*N*	24	24
	Mean [SD]	1.12 [0,80]	30.41 [23.14]
HDM Control	*N*	15	15
	Mean [SD]	0.77 [0.71]	22.49 [26.64]

Abbreviations: AIT, population of the AIT study; Control, population of the study with symptomatic medication only; CSMS, Combined Symptom and Medication Score; GP, grass pollen allergy patients; HDM, house dust mites allergy patients; SD, standard deviation; TP, tree pollen allergy patients; VAS overall, Visual Analogue Scale for overall impairment by symptoms.

**TABLE 5:** Values obtained by assessments during visits at the study centres

Pollen Season 2019/exposure period 2018		V1	V2		V3
Assessment		RQLQ	RQLQ	RCAT	RQLQ
TP AIT with 2 observations	*N*	30	30	28	30
	Mean [SD]	1.45 [1.01]	1.46 [0.89]	21.21 [4.44]	1.06 [0.99]
GP AIT with 2 observations	*N*	29	28	28	27
	Mean [SD]	0.60 [0.84]	1.47 [0.92]	20.96 [4.80]	1.15 [1.23]
HDM AIT with 2 observations	*N*	57	52	53	50
	Mean [SD]	1.94 [1.13]	1.63 [1.00]	20.83 [4.14]	1.61 [1.06]

Pollen season 2020/exposure period 2019		V1^b^ (V4^a^)	V2^b^ (V5^a^)		V3^b^ (V6^a^)
Assessment		RQLQ	RQLQ	RCAT	RQLQ
TP AIT with 2 observations	*N*	23	23	23	22
	Mean [SD]	0.95 [0.92]	1.38 [1.08]	21.78 [5.08]	1.07 [0.99]
TP AIT with single observation	*N*	15	14	14	14
	Mean [SD]	0.59 [0.79]	1.48 [1.58]	20.50 [7.53]	0.94 [0.88]
TP Control	*N*	8	8	8	8
	Mean [SD]	1.50 [1.46]	1.76 [1.24]	21.25 [3.28]	1.57 [1.25]
GP AIT with 2 observations	*N*	18	18	19	18
	Mean [SD]	1.42 [1.19]	2.50 [1.19]	18.68 [4.73]	1.36 [1.09]
GP AIT with single observation	*N*	11	11	11	11
	Mean [SD]	0.89 [1.16]	1.55 [0.92]	20.18 [5.53]	1.19 [0.64]
GP Control	*N*	33	32	32	32
	Mean [SD]	2.61 [1.28]	1.94 [1.14]	19.91 [4.67]	1.77 [1.05]
HDM with 2 observations	*N*	46	46	44	45
	Mean [SD]	1.15 [0.90]	1.39 [1.04]	22.14 [4.46]	1.05 [0.97]
HDM with single observation	*N*	24	23	23	23
	Mean [SD]	1.35 [0.94]	1.46 [0.96]	21.96 [2.88]	1.41 [0.80]
HDM Control	*N*	15	14	14	14
	Mean [SD]	0.60 [0.90]	0.71 [0.95]	25.57 [4.73]	0.48 [0.62]

Abbreviations: AIT, population of the AIT study; Control, population of the study with symptomatic medication only; GP, grass pollen allergy patients; HDM, house dust mites allergy patients; RCAT, Rhinitis Control Assessment Test; RQLQ, Standardised Rhinoconjunctivitis Quality of Life Questionnaire; SD, standard deviation; TP, tree pollen allergy patients.

^a^ AIT population patients with two assigned observations.

^b^ AIT population patients with single observation period and control population patients.

### 3.3 Comparison of seasons/exposure periods within the AIT population with two observations

In TP, mean CSMS slightly decreased, comparing PS 2019–2020. Mean VAS was similar in both seasons. Mean RQLQ score at the second visit (i.e., peak season) was slightly lower in the second season, while mean RCAT indicated controlled symptoms in both seasons. Surprisingly, in GP, an increase in CSMS, VAS and RQLQ was observed. Consistent with this, the RCAT score decreased, indicating worsened symptom control. In HDM, a decrease in mean CSMS, mean VAS and in mean RQLQ score at peak season was observed (17%, 21% and 15%, respectively). The mean RCAT score increased changing from uncontrolled to controlled symptoms.

### 3.4 Comparisons between the populations for pollen season 2020/exposure period 2019

In TP, mean CSMS was the lowest in AIT patients during their second observation period. Mean overall VAS score was similar in second observation AIT patients and control population, whereas it was higher in single observation AIT patients. Mean RQLQ score at the second visit was lower in AIT patients compared to control population patients. Mean RCAT scores were comparable, with second observation AIT patients' mean being closest to indicate controlled symptoms (>21).

In GP, CSMS between AIT and control population was comparable, and VAS only differed in AIT patients with a single treatment year. In the second observation period, GP AIT patients showed the highest RQLQ score, thus lowest QoL. All GP patients demonstrated uncontrolled symptoms as measured by RCAT, independent of their treatment duration.

In HDM patients, the control population showed better values regarding the assessments than the AIT population. Considering the first RQLQ score assessed after enrolment (i.e., V1 according to the observation plan), the HDM control population showed a distinctly lower, thus better RQLQ score compared to the HDM AIT patients, suggesting that patients enrolled in the control population and not taking AIT therapy had already been remarkably less affected at baseline.

### 3.5 Correlations between CSMS and validated questionnaires

The CSMS and RQLQ(S) correlation analyses were performed with the RQLQ score at V2 and V5, where available. In the AIT population, valid data sets were obtained from 149 patients for one observation period and 86 patients contributed to the calculation with second observation period data. Valid pairs of values of 54 patients were available in the control population. A positive correlation was shown in the AIT population with *r* = 0.426 (95% CI; 0.326; 0.583) as well as in the control population, with *r* = 0.569 (95% CI; 0.371; 0.920).

Analyses for CSMS and RCAT were performed with single observation data of 149 patients and second observation data of 85 in the AIT population. 54 patients from the control population contributed to the calculation. Negative correlation with *r* = −0.409 (95% CI; −0.56; −0.30) for AIT and *r* = −0.547 (95% CI; −0.88; −0.33) for control was shown, which was expected.

Correlation between CSMS and VAS was calculated for the AIT population with single observation data from 155 patients and with second observation data from 88. In the control population, data of 56 patients were included. Positive correlation between CSMS and VAS for overall impairment by ARC symptoms was shown with *r* = 0.585 (95% CI; 0.543; 0.796) in AIT and *r* = 0.563 (95% CI; 0.367; 0.906) in control.

**FIGURE 1:**
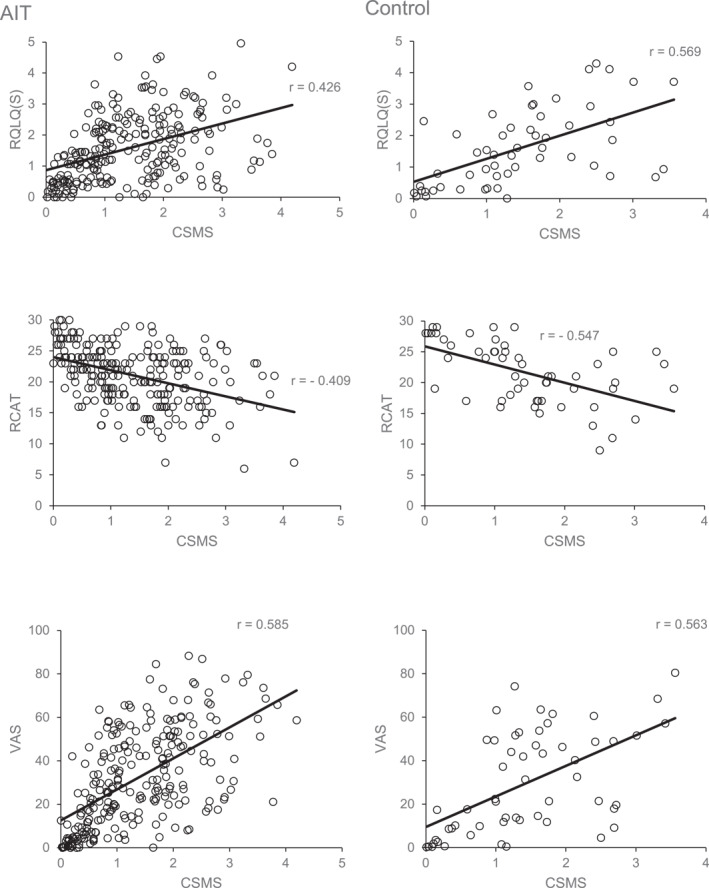
Correlation graphs. These graphs show the correlations between the combined symptom and medication score (CSMS) and each validated questionnaire used in the two populations. CSMS, Combined Symptom and Medication Score; RCAT, Rhinitis Control Assessment Test; RQLQ(S), Standardised Rhinoconjunctivitis Quality of Life Questionnaire; VAS, Visual Analogue Scale of overall impairment by allergic rhinoconjunctivitis symptoms

The correlations are depicted in Figure 1.

## 4 DISCUSSION

Because of the interdependence of symptoms and medication, the World Allergy Organization (WAO) recommended combined symptom and medication scoring as primary outcome measure.^31^ Clark and Schall^32^ showed that a combination of the Average Rhinoconjunctivitis Total Symptom Score (ARTSS) and Average Rescue Medication Score (ARMS) provided better discriminatory power than each of them alone. Grouin et al,^33^ suggested a refinement of the RTSS, the Adjusted daily Symptom Score (AdSS), which considers the effect of medication intake on symptom score with a last‐observation‐carried‐forward approach. A medication score is not added. A comparison between results obtained with a Combined Score (RTSS and RMS) and the AdSS showed that treatment effects were consistently demonstrated with both scores.^34^ However, it should be noted that medication scores add important information^33^ and contribute to the stepwise approach of medication rating as recommended by the WAO and consider the pharmacologically weighted impact of different medications on the symptom score.^13^, ^31^ Symptom and medication scoring can be done daily.^31^ Daily assessment is also possible using Visual Analogue Scales. VASs have been thoroughly investigated concerning their validity, especially with a mobile application more recently^21^, ^22^, ^23^, ^24^, ^35^ and the EAACI recommended it as a secondary outcome in AIT RCTs.^13^ They may also be particularly suitable in children.^36^ But VAS rating is clearly subjective^13^ and does not assess the use of rescue medication. In a recent study to evaluate correlations of a VAS referring to work impairment by allergic rhinitis, overall impairment VAS was implemented into the symptom score calculation of a modified CSMS.^35^ In the present study, the CSMS was used as recommended by the EAACI in its 2014 position paper.^13^ It was also used in recent studies on ultra‐short‐course booster SCIT^37^ and SLIT,^38^ reflecting the clinical effects of the therapy. In a 2019 review article, it was proposed to modify the medication score of the CSMS by deleting oral corticosteroids. Instead, the combined use of a corticosteroid and an antihistamine or a combination topical corticosteroid/antihistamine was proposed to score 3, with the occasional use of oral corticosteroid only being recorded but not included in the CSMS. The reason is that they are only taken very rarely in ARC trials which reduces the effective medication component being 0–2 instead of 0–3 as originally intended.^36^

To our knowledge, the present study is among the first to scrutinise a correlation between the CSMS and validated ARC assessments. Very recently, Sousa‐Pinto et al. elaborately reported on the validation of hypothesis‐driven and data‐driven CSMSs, based on large‐scale data, previously obtained with a widely used mobile app.^39^ Concurrent validity was analysed by comparison to other validated assessments than used in the present study, with respect to quality of life, impact on work and control of allergic diseases. In their discussion, the authors emphasize the need for prospective evaluations. Here, we analysed correlations by assessing the CSMS prospectively alongside the validated assessments on quality of life, symptom severity and symptom control. Our results show correlations as given by Pearson's *r*. Concerning their strength, they may be seen as moderate.^40^ With respect to the data shown in Figure 1 and calculating the coefficient of determination (*R* ^2^), ranging from 0.17 to 0.34, the linearity between CSMS and the validated questionnaires is limited.^41^ This likely is the result of the different methodology and endpoints of the CSMS that does not equally assess how and what the other questionnaires assess. For instance, the RQLQ(S), assesses nasal and ocular symptoms, but additionally covers other domains with different subsets of questions, contemplating 7 days, retrospectively. The VAS assesses perceived impairment by ARC symptoms on a continuous scale rather than in terms of categorical endpoints^42^ like the CSMS does. The RCAT evaluates rhinitis control based on a 1‐week recall period for each item^43^ with the answering options for five out of six questions, including symptoms, being categories of frequency rather than severity as assessed by the CSMS. In contrast, the latter is a daily assessment and, being a central feature, incorporates the intake of medication, thus adding relevant information which is not assessed by the other questionnaires at all. The correlation coefficients for CSMS and VAS were comparable between both study populations, and regarding the AIT population, this correlation was the strongest. Accordingly, the *R* ^2^‐value was the comparatively highest. In our view, this may be since both values were assessed daily, whereas RQLQ(S) and RCAT had longer recall periods and were only recorded during the visits. Both CSMS and VAS share a concept of focusing on symptom severity or impairment by symptoms. Furthermore, we think, it reflects that medication intake impacts the impairment by ARC symptoms.

Our study has several limitations. One is given by the inherently heterogenous data quality. The number of CSMS diary entries varied from patient to patient. While some patients filled in the diary completely and regularly, others did not complete the requested 30 daily entries and/or filled in the diary irregularly and/or did not properly mind the pollen seasonality. By only analysing the data that were harmonised with the respective pollen season or worst symptom load for HDM, we were able to compensate for this limitation. Further, whereas the RQLQ(S) was answered at 3 time‐points of the season/exposure period, the RCAT was only assessed once, thus limiting the overall interpretation. These questionnaires were filled in retrospectively contemplating the last week, which might have affected accuracy of outcomes. In contrast, the CSMS was filled in daily, possibly favouring its use since less information is likely to be missed out compared with retrospective assessments. Due to the observational design of our studies, visits were not always carried out according to the observation plan but rather followed the daily practice routine which itself could have negatively influenced the outcome parameters and the correlation analysis. The study design, including the different size of the groups, must also be borne in mind, when looking at the comparisons of the outcome parameters. On the other hand, however, this daily practice routine data generated by our investigation show correlations between the CSMS and validated questionnaires. The EAACI emphasized the need for CSMS validation to be carried out in multicentre, multinational trials.^13^ In that sense, although not being multinational, another strength of our studies was their multicentre design, covering different areas of Germany, with three different allergy groups and two therapy modes. However, comprehensive validation of the CSMS should be attempted in multinational, well powered phase III studies, as appealed for.^13^, ^15^ We think, our results essentially contribute to discussions, supporting the view that the CSMS is a valuable instrument to be used as clinical endpoint in ARC.

## AUTHOR CONTRIBUTIONS

Binoy Palathumpattu: Data curation; Lead, Formal analysis; Supporting, Project administration; Supporting, Writing – original draft; Lead, Writing – review & editing; Lead, Ursula Pieper‐Furst: Conceptualization; Supporting, Project administration; Lead, Writing – review & editing; Equal, Cengiz Acikel: Formal analysis; Equal, Writing – review & editing; Supporting, Hacer Sahin: Formal analysis; Equal, Writing – review & editing; Supporting, Silke Allekotte: Conceptualization; Equal, Writing – review & editing; Equal, Jaswinder Singh: Software; Lead, Writing – review & editing; Supporting, Mark Wilhelm Hess: Conceptualization; Equal, Project administration; Supporting, Writing – review & editing; Equal, Angelika Sager: Conceptualization; Supporting, Resources; Equal, Supervision; Supporting, Writing – review & editing; Supporting, Thomas Muller: Conceptualization; Supporting, Resources; Equal, Supervision; Supporting, Writing – review & editing; Supporting, Ralph Mosges: Conceptualization; Lead, Funding acquisition; Lead, Supervision; Lead, Writing – review & editing; Equal

## CONFLICT OF INTEREST

This research was funded by LETI Pharma GmbH, Ismaning, Germany. Angelika Sager and Thomas Müller are employees of LETI Pharma. Ralph Mösges reports grants, personal fees and non‐financial support from LETI Pharma during the conduct of the study; personal fees from ALK, grants from ASIT biotech, personal fees from Allergopharma, personal fees from Allergy Therapeutics, grants and personal fees from Bencard, grants, personal fees and non‐financial support from Lofarma, non‐financial support from Roxall, grants and personal fees from Stallergenes, grants from Optima, personal fees from Friulchem, personal fees from Hexal, personal fees from Servier, personal fees from Klosterfrau, non‐financial support from Atmos, personal fees from Bayer, non‐financial support from Bionorica, personal fees from FAES, personal fees from GSK, personal fees from MSD, personal fees from Johnson and Johnson, personal fees from Meda, personal fees and non‐financial support from Novartis, non‐financial support from Otonomy, personal fees from Stada, personal fees from UCB, non‐financial support from Ferrero, grants from bitop AG, grants from Hulka, personal fees from Nuvo, grants and personal fees from Ursapharm, personal fees from Menarini, personal fees from Mundipharma, personal fees from Pohl‐Boskamp, grants from Inmunotek, grants from Cassella‐med GmbH and Co. KG, personal fees from Laboratoire de la Mer, personal fees from Sidroga, grants and personal fees from HAL BV, personal fees from Lek, personal fees from Pro‐AdWise, personal fees from Angelini Pharma, outside the submitted work. Ursula Pieper‐Fürst, Cengizhan Acikel, Hacer Sahin, Silke Allekotte, Jaswinder Singh, Mark Hess and Binoy Palathumpattu have no conflicts of interest to declare.
